# Shift in the metabolic profile of sediment microbial communities during seagrass decline

**DOI:** 10.1186/s40793-025-00750-1

**Published:** 2025-07-22

**Authors:** Marsej Markovski, Mirjana Najdek, Zihao Zhao, Gerhard J. Herndl, Marino Korlević

**Affiliations:** 1 Centre for Marine Research, Ruđer Bošković Institute, G. Paliaga 5, 52210 Rovinj, Croatia; 2 Department of Functional and Evolutionary Ecology, University of Vienna, Djerassiplatz 1, 1030 Vienna, Austria; 3 Department of Marine Microbiology and Biogeochemistry, Royal Netherlands Institute for Sea Research (NIOZ), Utrecht University, PO Box 59, 1790 AB Den Burg, The Netherlands

**Keywords:** Sediment microbial communities, *Cymodocea nodosa*, Seagrass meadow decline, Northern Adriatic Sea, Metaproteomics, Microbial metabolic profile

## Abstract

****Background**:**

Seagrass meadows are highly productive ecosystems that are considered hotspots for carbon sequestration and microbial activity. In seagrass sediments, microbial communities break down organic matter, facilitating the release and transformation of nutrients that support plant growth and primary production. The decline of seagrass meadows of various species has been documented worldwide, including that of *Cymodocea nodosa* (Ucria) Ascherson, a widespread seagrass in the Mediterranean Sea. To assess the influence of seagrass decline on the metabolic profile of sediment microbial communities, metaproteomes from two sites, one without vegetation and one with a declining *Cymodocea nodosa* meadow, were characterised at monthly intervals from July 2017 to October 2018.

****Results**:**

Prior to seagrass decline, differences in the metabolic profiles between the vegetated and nonvegetated sediment were found, particularly in the deeper sediment layers. During the decline, these differences diminished as microbial communities in nonvegetated sediments exhibited increased protein richness and diversity, aligning more closely with those at the vegetated site. Notably, temporal variations in the structure of the metabolic profile were only observable in the nonvegetated sediment and were also more pronounced at greater sediment depths. Finally, the assessment of proteins involved in organic matter degradation such as ABC transporters, fermentation-mediating enzymes, and proteins involved in dissimilatory sulphate reduction mirrored these shifts.

****Conclusions**:**

Overall, the main results of this study suggest that the presence of seagrass meadows influences the metabolic profile of microbial communities in sediments, highlighting the distinctions between nonvegetated and seagrass-colonised sediments. In particular, the loss of seagrass leads to a shift in the metabolic profile of sediment communities in the surrounding area, while the metabolic profiles of previously colonised sediments appear to be more resilient to seagrass loss.

## Background

The biomass in marine sediments consists mainly of prokaryotes, whose richness and abundance are comparable to that in the water column [1–3]. The main factor determining the abundance and activity of these microorganisms is the availability of organic matter [3–5]. The complete mineralisation of organic compounds in anoxic environments such as most coastal sediments requires complex microbial interactions [6–9]. The stepwise degradation of organic matter begins with the breakdown of complex organic polymers such as carbohydrates or proteins by extracellular enzymes that can be released into solution or remain associated with the cell. These enzymes convert high-molecular-weight organic matter to substrates that are small enough to be transported into the cell [10]. Part of these hydrolytic products are fermented to short-chain fatty acids (SCFAs) and alcohols that facilitate anaerobic microbial respiration, e.g. by sulphate-reducing bacteria or methanogens [7, 9].

Shallow coastal sediments colonised by seagrasses are considered a special type of habitat where microbes break down organic matter, facilitating the release and transformation of nutrients that support plant growth and primary production [11]. Such areas are hotspots for microbial activity, as seagrasses enrich the sediment with organic matter by excreting organic carbon, trapping organic particles from the water column, and stabilising the sediment. In addition, the decomposition of seagrass leaves, roots, and rhizomes contributes to the enrichment of the sediment with organic matter [11–14]. Consequently, microbial communities in seagrass sediments are metabolically more diverse and active than those inhabiting bare sediments [15]. Taxonomic analyses showed differences between communities at vegetated and nonvegetated sites [16–21] and indicated that microbial communities even differ with respect to the meadow edge [22].

In order to obtain a comprehensive overview of the microbial communities living in sediments colonised by seagrasses, methods that allow functional characterisation, such as metaproteomics, must be applied. This high-throughput “meta-omics” approach is emerging as an important tool for deciphering the key components that determine the function of microbial ecosystems [23]. In addition, this approach has the potential to provide insights into the biogeochemical cycling in marine sediments and to assess the response of microbes to environmental change [24]. Metaproteomics is closely linked to metagenomics, as genome information in combination with data on expressed proteins not only provides information on the functional potential of microbial populations, but also on which metabolism is active in an ecosystem [25]. Metaproteomics has already been used to analyse microbial metabolic processes in cold seeps [26, 27], diffuse hydrothermal venting [28], mudflat aquaculture [29], and chronically petroleum-polluted [30] sediments, but to our knowledge there are no metaproteomic studies on microbial communities in seagrass meadow sediments.

About 19% of seagrass meadows worldwide have been lost since 1880 [31]. A decline of *Cymodocea nodosa* (Ucria) Ascherson, a widespread and common seagrass species throughout the Mediterranean Sea [32], has been observed [33–36], including in the northern Adriatic Sea [37–39]. However, there is little information on microbial dynamics during seagrass decline, making it difficult to predict how the loss of seagrass influences the microbial community in the sediment. The few available studies on microbial community succession in seagrass sediments suggest that changes could be expected. For instance, changes in sulphate-reducing bacteria in seagrass bed sediments over time were reported [40], as well as community changes in response to nutrient availability [41] and seagrass restoration [42]. However, in a previous study, we investigated the diversity and dynamics of sediment microbial communities during the decline of the seagrass species *C. nodosa* and found a notable compositional stability in response to such a major disturbance [21]. The aim of the present study was to characterise the metabolic profile of prokaryotic communities in *C. nodosa* meadow sediments using a metaproteomic approach, with the hypotheses that: (i) there are differences in metabolic profiles between vegetated and nonvegetated sediments that vary with sediment depth, and (ii) the decline of seagrass meadows leads to a shift in the microbial metabolic profile that is not uniform across sediment layers.

## Methods

### Sampling

Sampling for DNA and protein isolation was performed as described in Markovski et al. (2022) [21]. Briefly, sediment samples were collected in a declining *C. nodosa* meadow (vegetated site) in the Bay of Saline, a shallow and dynamic coastal area 4 km north-west of Rovinj, Croatia, on the east coast of the northern Adriatic Sea (45°7′ 5″ N, 13°37′ 20″ E). An adjacent area without seagrass (nonvegetated site) was also sampled in the same bay. From July 2017 to October 2018, a sediment core was taken monthly at each site by diving with plastic core samplers. As seagrass surface sediments show vertical patterns of environmental conditions [39] and microbial community structures [19], the sediment cores were cut into four sections of 1 cm length each: the top (0–1 cm), the bottom (7–8 cm), and two middle sections: upper middle (2–3 cm) and lower middle (3–6 cm; Supplementary Table S1). A detailed description of the sampling site, the environmental conditions, and the decline of the *C. nodosa* meadow can be found in Najdek et al. (2020) [39]. In brief, at the beginning of the study, part of the bay was covered with a large and dense seagrass meadow extending from the south-western coastal area towards the central part of the bay. The seagrass showed a regular growth minimum in November 2017. After that, the shoots and leaves began to decline, while roots and rhizomes persisted longer, until March 2018. At the end of the study, only small patches of the meadow were still present in the form of tiny strips along the shoreline [39]. The decline of the meadow was attributed to the reduced light availability caused by the increased turbidity of the seawater due to the increased terrigenous input [39].

### DNA isolation

Total DNA from each sediment section was isolated using a modified isolation protocol [43] based on Zhou et al. (1996) [44] as described in Markovski et al. (2022) [21]. In brief, 2 g of sediment were weighed, avoiding roots and rhizomes in vegetated cores, mixed with the extraction buffer and proteinase K, and incubated by horizontal shaking at 37 °C for 30 min. After the addition of SDS, the mixture was incubated again by horizontal shaking at 65 °C for 60 min. The sediment particles were removed by centrifugation and the supernatant was extracted three times with an equal volume of chloroform:isoamyl alcohol (1:1). DNA precipitation was performed by adding isopropanol and incubating the mixture at 22 °C for 60 min. The DNA pellet obtained after the centrifugation step was washed twice with cold (−20 °C) 70% ethanol, air-dried, and resuspended in 100 µl of deionised water.

### Metagenomics

Due to the limited number of sediment metagenomes that could have been sequenced, we selected four DNA samples from Markovski et al. (2022) [21] collected in August 2018 from the top (0–1 cm) and lower middle (4–5 cm) layers of both the vegetated and nonvegetated sites (Supplementary Table S2). These selected DNA samples were sent on dry ice to IMGM Laboratories (Martinsried, Germany) for metagenomic sequencing. The genomic DNA was purified using AMPure XP Beads (Beckman Coulter, USA) at a bead:DNA ratio of 1:1 (v/v) and quantified using the Qubit dsDNA HS Assay Kit (Thermo Fisher Scientific, USA). The integrity of the DNA was checked on a 1% agarose gel. Metagenomic sequencing libraries were prepared from 100 or 300 ng of genomic DNA using the NEBNext Ultra II FS DNA Library Prep Kit for Illumina (New England Biolabs, USA) according to the manufacturer’s protocol. Fragments of 500–700 bp were selected using the AMPure XP Beads, enriched by PCR for 5 or 6 cycles, and quality controlled. The individual libraries generated from different DNA input samples were pooled and sequenced on an Illumina NovaSeq 6000 sequencing system (2 × 250 bp).

The sequences obtained were analysed on the Life Science Compute Cluster (LiSC; CUBE—Computational Systems Biology, University of Vienna). MEGAHIT (version 1.2.9) [45], with default settings, was used to assemble individual metagenomic libraries and putative genes were predicted from contigs longer than 200 bp using Prodigal (version 2.6.3) [46] in metagenome mode (-p meta). Predicted genes were functionally annotated using the eggNOG mapper (version 2.1.9) [47] with the eggNOG database (version 5.0.2) [48]. Taxonomic classification was performed using the lowest common ancestor algorithm from DIAMOND (version 2.0.15) [49] against the non-redundant NCBI database (NR). Phylogeny was determined using the top 10% of hits with an e-value < 1 × 10^-5^ (--top 10). Sequence renaming and the calculation of metagenomic statistics were performed using the tools from BBTools (https://jgi.doe.gov/data-and-tools/bbtools). In total, metagenomic sequencing generated between 205,085,833 and 216,556,629 sequence pairs (Supplementary Table S2). After the removal of low-quality reads, sequences were assembled into 21,634,340 to 33,248,196 contigs, with L50 ranging from 590 to 601 bp. Coding sequence (CDS) prediction generated between 27,526,969 and 42,249,295 CDSs, while functional annotation resulted in 19,599,377 to 29,892,039 annotated CDSs.

### Protein isolation

The proteins were isolated from the same sediment sections that were used for DNA isolation [21]. For each of the 15 sampling dates, one protein sample was isolated for each of the four sections collected from the vegetated and nonvegetated site, resulting in a total of 120 protein samples (Supplementary Table S1). The SDS-based lysis method with trichloroacetic acid (TCA) precipitation described in Chourey et al. (2010) [50] and modified by Hultman et al. (2015) [51] was used. To 5 g of sediment, 10% (w/w) polyvinylpolypyrolidone (PVPP) was added. The mixture was suspended in 5 ml protein extraction buffer (4% SDS; 100 mM Tris–HCl, pH 8.0) and vortexed. After incubation in boiling water for 5 min, the samples were sonicated and incubated again in boiling water for 5 min. Sonication was performed using the Sonopuls HD 4100 probe sonicator (Bandelin, Germany) equipped with an UW 100 ultrasonic transducer and a TS 103 probe. The solution was sonicated at 75% of the maximum amplitude (245 μm) for 2 min at an interval of 10 s on and 10 s off. The sediment particles were removed by centrifugation for 20 min at 4 °C and 4,500 × *g*. The supernatant was transferred to a clean tube and mixed with 1 M dithiothreitol (DTT; final concentration 24 mM). The proteins were precipitated with cold (4 °C) 100% TCA (final concentration 20%) overnight at −20 °C. The protein pellet was obtained by centrifugation for 40 min at 4 °C and 10,000 × *g*. The obtained pellet was washed three times with cold (−20 °C) acetone and centrifuged after each washing step for 5 min at 4 °C and 20,000 × *g*. The pellet was transferred to a clean 1.5 mL tube during the first washing step. The dried pellet was stored at −80 °C until further processing.

### Metaproteomics

The filter-aided sample preparation (FASP) [52] procedure was used to perform trypsin digestion. Isolated proteins were processed using the FASP Protein Digestion Kit (Expedeon, UK) according to the manufacturer’s instructions, with minor modifications [53]. Briefly, the protein pellet was solubilised in the urea sample buffer included in the kit, amended with DTT, and centrifuged to remove larger particles. The trypsin digestion was performed on the column filter overnight at 37 °C for 18 h. The resulting filtrate containing peptides was acidified to a final concentration of 1% trifluoroacetic acid (TFA). Digested peptides were desalted using the Pierce C18 Tips (Thermo Fisher Scientific, USA) according to the manufacturer’s instructions and sent to the Proteomics Facilities of the University of Vienna for mass spectrometry analysis.

MS/MS spectra were obtained using a Q Exactive Hybrid Quadrupole-Orbitrap Mass Spectrometer (Thermo Fisher Scientific, USA) and searched against a protein database containing amino acid sequences of predicted CDSs from combined metagenomes that were sequenced and analysed as described above. MS/MS spectra were successfully generated for 118 samples (Supplementary Table S1). Before the protein database search, the predicted CDSs were clustered at 90% similarity using CD-HIT (version 4.6.8). Peptides were identified using the SEQUEST-HT engine and validated with Percolator, all within Proteome Discoverer (version 2.1; Thermo Fisher Scientific, USA). The probability of false peptide identification was reduced by applying the target-decoy approach. Only peptides with a false discovery rate < 1% were retained. Protein identification required at least two peptides, including one unique peptide. Quantification of the relative abundance of proteins was conducted using a chromatographic peak area-based label-free quantitative method [54, 55], where the peak areas of unique peptides were summed and normalised to the normalised area abundance factor (NAAF). In total, 67,947 different proteins were identified from the obtained MS/MS spectra. Of all identified proteins, 94.6% were annotated by the eggNOG database. To focus exclusively on microbial communities, only proteins classified as *Archaea* and *Bacteria* using the NCBI database were retained. As a precautionary measure, proteins that were not taxonomically classified as *Archaea* or *Bacteria* by the eggNOG database were also removed, leaving a total of 57,305 proteins in the dataset.

### Data analysis

Data processing and visualisation were performed using R (version 4.5.0) [56] combined with the tidyverse package (version 2.0.0) [57, 58] and several other packages [59–78]. The Shannon diversity index was calculated using the function diversity from the vegan package (version 2.6.10) [71]. To express the diversity index in terms of the effective number of proteins, the exponential of the Shannon diversity index was calculated [79]. Differences between the number of observed proteins, the exponential of the Shannon diversity index, and the NAAFs between sites, sediment layers, i.e. sections of sediment cores, and the period before and during the decline of the *C. nodosa* meadow were tested by applying the Mann–Whitney *U* test using the function wilcox.test [56]. The Bonferroni correction was applied to solve the problem of multiple comparisons using the function p.adjust [56]. Differences in the structure of the microbial metabolic profiles between sites, sediment layers, and the period before and during the meadow decline were tested on Bray–Curtis dissimilarities based on protein NAAFs by performing the Analysis of Similarities (ANOSIM) using the function anosim from the vegan package and 999 permutations [71]. The grouping of samples into the period before and during meadow decline was based on the status assessment of the *C. nodosa* meadow reported by Najdek et al. (2020) [39], focusing on the belowground biomass. The sampling period from the beginning of the study until February 2018 was labelled the period before decline, while the period after this month was referred to as the period of meadow decline. Principal Coordinate Analysis (PCoA) was performed on Bray–Curtis dissimilarities based on protein NAAFs using the function wcmdscale from the vegan package. If necessary, the Lingoes correction method was applied to account for negative eigenvalues [71, 80, 81].

## Results

**Fig. 1:**
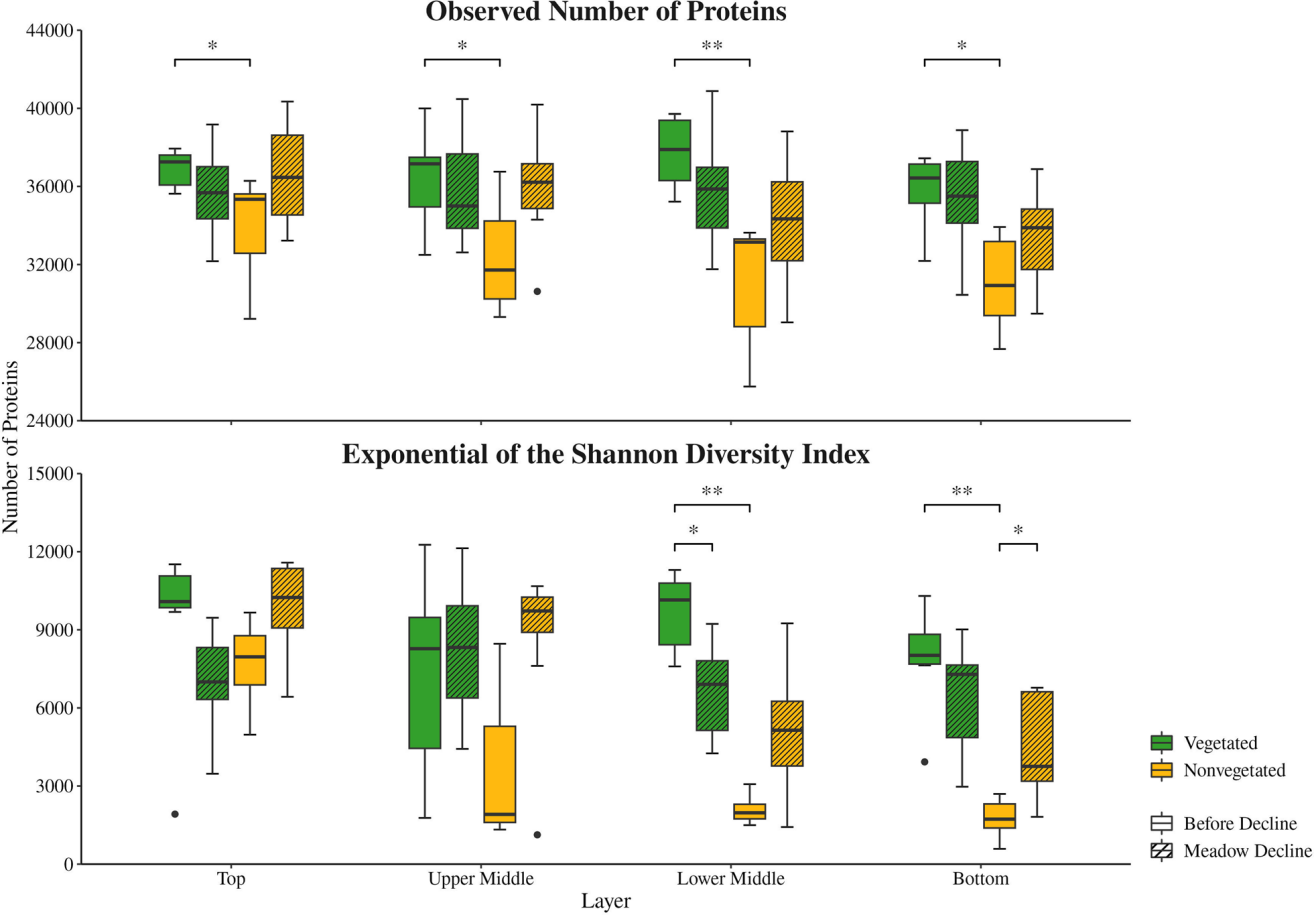
The observed number of proteins and the exponential of the Shannon diversity index of sediment microbial communities in the Bay of Saline. Samples were collected in different sediment layers at the vegetated and nonvegetated site before and during the decline of the *C. nodosa* meadow. The asterisks indicate the level of statistical significance: *p < 0.05 and **p < 0.01, while the dots represent outliers

To assess the richness and diversity of isolated proteins from the sediment microbial communities in the Bay of Saline, the number of observed proteins and the exponential of the Shannon diversity index were calculated. Samples from each layer were grouped based on the sampling site and the period before and during the meadow decline. Comparisons were made both between sampling sites within each period and between different periods at each individual site (Fig. 1). In all layers, significantly (*p* < 0.05) higher numbers of observed proteins were found during the period before meadow decline at the vegetated (top, 35,626–37,937 proteins; upper middle, 32,494–39,996 proteins; lower middle, 35,220–39,713 proteins; and bottom, 32,183–37,440 proteins) compared to the nonvegetated (top, 29,217–36,284 proteins; upper middle, 29,312–36,755 proteins; lower middle, 25,752–33,630 proteins; and bottom, 27,672–33,922 proteins) site. In contrast, no significant changes between sites were observed in all layers during the period of decline. In addition, no significant changes were found at each site between the two periods. When analysing the exponential of the Shannon diversity index, significant changes were observed only in the lower middle and bottom layer (Fig. 1). Here, in agreement with the number of observed proteins, higher values were found during the period before meadow decline at the vegetated (lower middle, 7,594.2–11,300.0 proteins and bottom, 3,927.3–10,300.9 proteins) compared to the nonvegetated site (lower middle, 1,497.2–3,070.7 proteins and bottom, 586.6–2,696.5 proteins). Also, in agreement with the number of observed proteins, no significant changes were observed between the sites during meadow decline. Additionally, the Shannon diversity index showed significant changes in these layers between the two periods. In the lower middle layer of the vegetated site, significantly higher values were observed before (7,594.2–11,300.0 proteins) than during (4,254.3–9,227.5 proteins) meadow decline. However, in the bottom layer of the nonvegetated site significantly higher values were found during (1,815.8–6,775.9 proteins) than before the meadow decline (586.6–2,696.5 proteins).

**Fig. 2:**
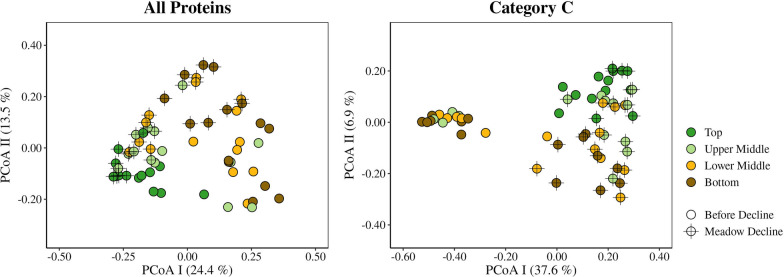
PCoA of Bray–Curtis dissimilarities of microbial proteins sampled in the sediment of the Bay of Saline. All proteins and proteins classified only into COG category C (energy production and conversion) were analysed. Only samples collected in sediment layers at the nonvegetated site before and during the decline of the *C. nodosa* meadow are shown. The proportion of variation explained by each axis is indicated in parentheses on the corresponding axis

**Fig. 3:**
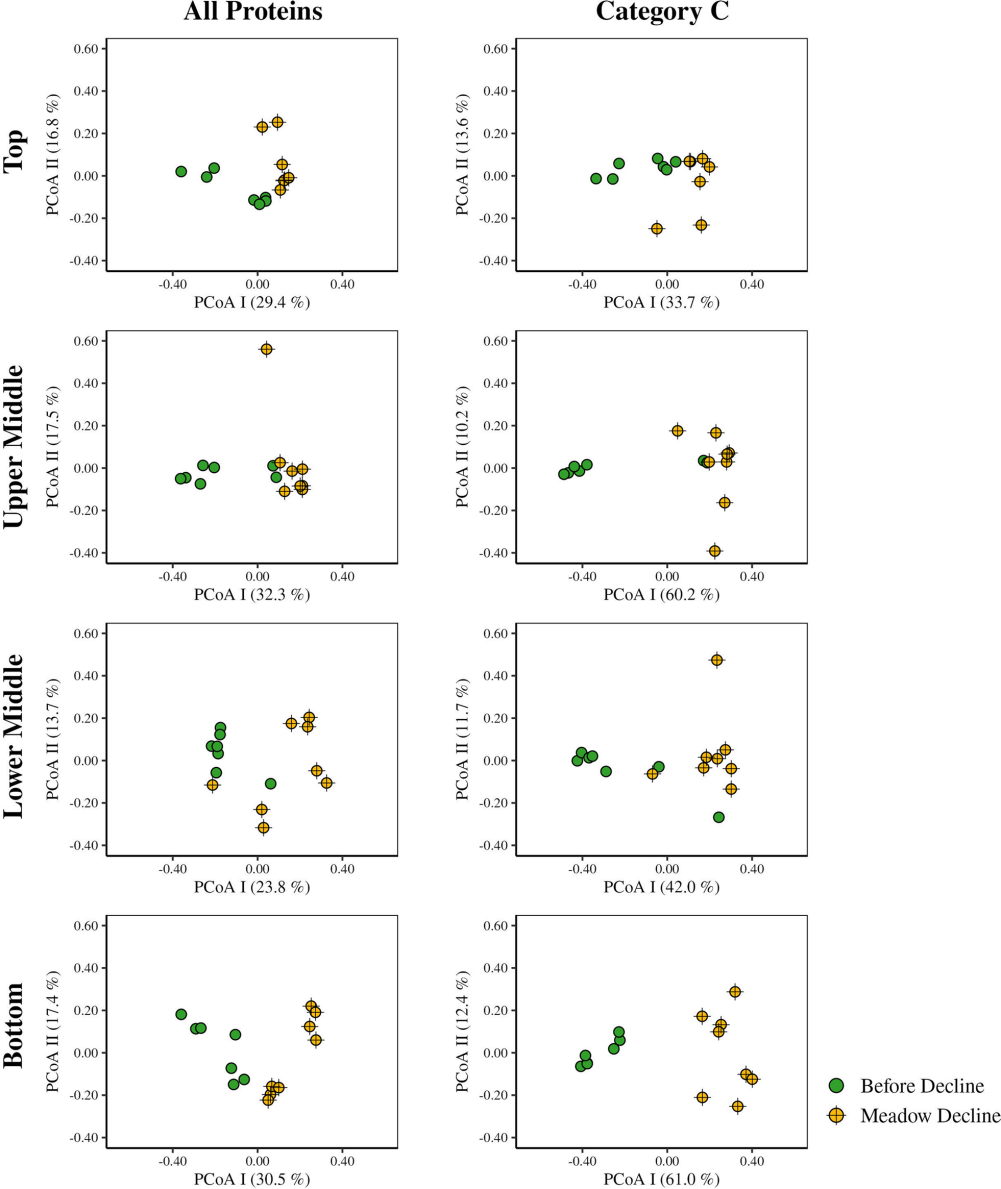
PCoA of Bray–Curtis dissimilarities of microbial proteins sampled in each layer in the sediment of the Bay of Saline. All proteins and proteins classified only into COG category C (energy production and conversion) were analysed. Only samples collected at the nonvegetated site before and during the decline of the *C. nodosa* meadow are shown. The proportion of variation explained by each axis is indicated in parentheses on the corresponding axis

ANOSIM testing of Bray–Curtis dissimilarities was applied to determine the changes in the structure of the metabolic profile of the sediment microbial communities. When all proteins from all samples were analysed together, no strong differentiation was observed between sites, layers, or the period before and during meadow decline (ANOSIM, R = 0.14–0.24, all *p* < 0.01). To determine whether only a part of the metabolic network showed any differentiation, proteins classified in the Cluster of Orthologous Genes (COG) category C (energy production and conversion), the most abundant category in our samples (see below, Supplementary Table S3), were analysed separately. However, no strong differentiation was observed between sites, layers, or decline periods when only these proteins were considered (ANOSIM, R = 0.15–0.21, all *p* < 0.01). In addition, the separate analysis of samples from the vegetated and nonvegetated site of all and COG C categorised proteins did also not reveal a strong differentiation between layers or decline periods (ANOSIM, R = 0.09–0.26, all *p* < 0.01), with the exception of a more pronounced separation observed at the nonvegetated site between the periods before and during meadow decline. This separation could be observed when all proteins (ANOSIM, R = 0.33, *p* < 0.01) and, especially, when proteins from the functional COG category C (ANOSIM, R = 0.51, *p* < 0.01) were considered. To gain a clearer overview of this separation, samples from the nonvegetated site were analysed using PCoA (Fig. 2). A distinction of samples from the lower middle and bottom layer retrieved during the period before meadow decline from all other samples was noticed. This distinction could be observed when all proteins were analysed together, but especially when proteins from the functional COG category C were considered (Fig. 2). Furthermore, to gain a better insight in the change of the structure of the metabolic profile between the period before and during meadow decline, samples from each site and layer were analysed separately. Sediment layers of the vegetated site did not show any strong differentiation between these two periods when either all (ANOSIM, R = 0.05–0.31, *p* = 0.01– 0.24) or only COG C categorised proteins (ANOSIM, R = 0.12–0.30, *p* = 0.01–0.07) were considered. In contrast, a pronounced separation between the two periods was observed in different layers of the nonvegetated site (Fig. 3). When comparing the layers of this site, the lowest distinction between the two periods was observed in the top layer (ANOSIM; all proteins, R = 0.35, *p* < 0.01; COG C proteins, R = 0.38, *p* < 0.01), middle in the upper and lower middle layer (ANOSIM; all proteins, R = 0.29–0.45, all *p* < 0.01; COG C proteins, R = 0.53–0.62, all *p* < 0.01), and the highest in the bottom layer (ANOSIM; all proteins, R = 0.53, *p* < 0.01; COG C proteins, R = 0.95, *p* < 0.01).

A total of 52,270 different proteins were assigned to a COG functional category. The most abundant COG category in terms of the number of proteins it contained (8,224 proteins) and their NAAFs (15.2%) was the functional COG category C, which comprises proteins for energy production and conversion (Supplementary Table S3). To detect how the decline of the meadow affected the energy production and conversion of sediment microbial communities in the Bay of Saline, we assessed the NAAF dynamics of the functional COG category C in each sediment layer. When comparing the sites before the meadow decline, significant (*p* < 0.05) differences were only observed in the bottom layer where the proteins of this functional category comprised a larger proportion at the nonvegetated (19.5–31.7%) than at the vegetated (13.8–26.2%) site. No significant difference was found between the sites during the decline. When comparing the layers of the individual sites before and during the decline, we detected a significant change in the proportion of the functional COG category C only in the bottom layer of the nonvegetated site. Here, a significant decrease in the proportion of this functional category was observed between the period before (19.5–31.7%) and during (8.2–13.9%) meadow decline.

**Fig. 4:**
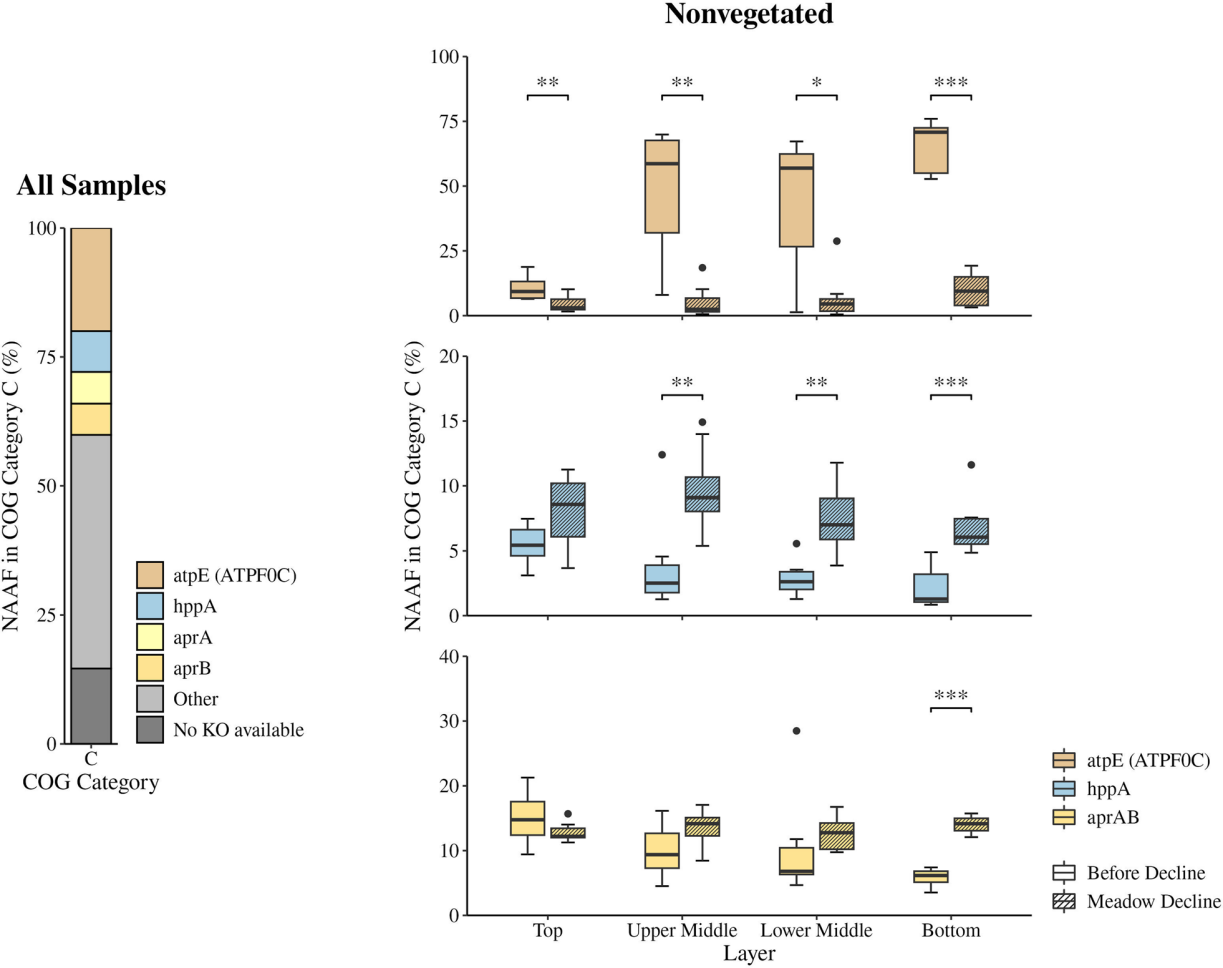
Proportion of the most abundant (> 3%) KEGG KO entries within the functional COG category C (energy production and conversion) in all samples and changes in the proportion of the same entries in each layer at the nonvegetated site before and during the decline of the *C. nodosa* meadow in the Bay of Saline. The proportion was calculated using the NAAF. The asterisks indicate the level of statistical significance: *p < 0.05, **p < 0.01, and ***p < 0.001, while the dots represent outliers

As the COG categories provide only a broad overview, the predicted CDSs were also classified using the Kyoto Encyclopaedia of Genes and Genomes (KEGG) Orthology (KO) database to gain better insight into the metabolic profile. A total of 1,408 different KO entries were present in the dataset, while 37,243 proteins were assigned to one or more of these KO entries. As the functional COG category C was the most abundant in our dataset (Supplementary Table S3), we aimed to further explore the dynamics of the most pronounced KO entries within this category. The F-type H^+^-transporting ATPase subunit c (ATPF0C, atpE; 20.0%), the K^+^-stimulated pyrophosphate-energised sodium pump (hppA; 7.9%), and the adenylylsulphate reductase subunits A (aprA; 6.2%) and B (aprB; 6.1%) represented the highest proportion (NAAF) within the functional COG category C (Fig. 4). As the samples from the nonvegetated site showed a clear separation based on the decline periods, especially when the COG category C dataset was considered (Fig. 2), we compared the proportion of these three proteins at the nonvegetated site before and during meadow decline (Fig. 4). We observed a significant (*p* < 0.05) decrease in the proportion of the F-type H^+^-transporting ATPase subunit c during the decline in all layers. This decrease was particularly pronounced in the bottom layer, where this protein constituted between 52.7 and 76.0% of all COG C categorised proteins before the decline. During meadow decline, its proportion dropped to between 3.2 and 19.3%. Although not as pronounced as the change in the F-type H^+^-transporting ATPase subunit c, the K^+^-stimulated pyrophosphate-energised sodium pump also showed a significant shift between the two periods in all layers, except the top layer. The most significant shift was observed in the bottom layer, where this protein increased from between 0.8 and 4.9% of all COG categorised proteins before the decline to between 4.9 and 11.6% during meadow decline (*p* < 0.001). The proportion of the adenylylsulphate reductase subunits A and B also increased during the decline in all layers, with the exception of the top layer. However, this shift was only significant in the bottom layer, where this protein increased from 3.5 to 7.4% of all COG categorised proteins before the decline to 12.1 to 15.7% during meadow decline.

**Fig. 5:**
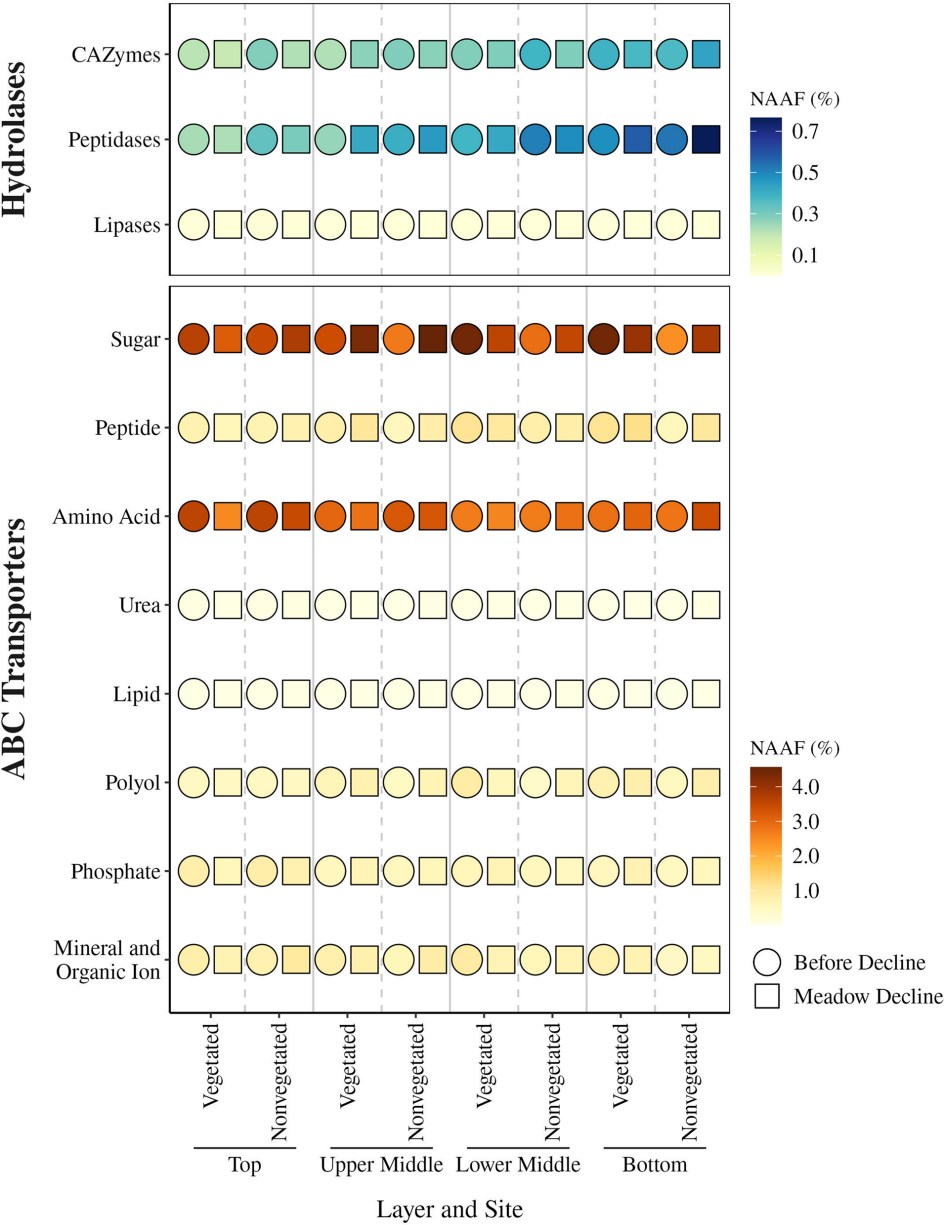
Median proportion of groups of hydrolases and ABC transporters in sediment layers at the vegetated and nonvegetated site before and during the decline of the *C. nodosa* meadow in the Bay of Saline. The proportion was calculated using the NAAF

The degradation of complex organic matter by the sediment microbial community in the Bay of Saline was evaluated by assessing the dynamics of the carbohydrate, protein, and lipid hydrolytic enzymes (Fig. 5). The dynamics of carbohydrate hydrolytic enzymes was determined using Carbohydrate-Active enZymes (CAZymes), whose proportion did not significantly (*p* < 0.05) change between sites or decline periods, with the exception of the top sediment layer at the nonvegetated site. Here, a significant decrease in the proportion of CAZymes was observed from the period before decline (0.28–0.43%) to the period of meadow decline (0.22–0.28%; Fig. 5). Proteins assigned to the glycoside hydrolase families GH5 and GH9 were the most abundant of all CAZymes (47.2%). To assess protein degradation, we focused on proteins assigned as peptidases in KEGG. The proportion of these enzymes significantly increased in the upper middle layer of the vegetated site from the period before decline (0.15–0.45%) to the period of meadow decline (0.34–0.61%; Fig. 5). Peptidases were almost exclusively comprised of metalloendopeptidases and serine endopeptidases (93.3%). Compared to CAZymes and peptidases, lipases were the least represented in our data (Fig. 5).

We assessed the dynamics of ATP-binding cassette (ABC) transporters to evaluate the uptake of hydrolytic products by prokaryotic cells. Substrate-binding proteins classified as ABC transporters in the KEGG Pathway (map02010) were selected and further manually classified into the following categories based on the molecules they transport: sugar, peptide, amino acid, urea, lipid, polyol, phosphate, and mineral and organic ion (Fig. 5). Sugar (38.0%) and amino acid (31.5%) transporters were the most abundant among all selected ABC transporters. In the lower middle and bottom layer, a significantly (*p* < 0.05) higher proportion of sugar ABC transporters was observed at the vegetated (lower middle, 2.90–4.57%; bottom, 3.90–5.52%) than at the nonvegetated (lower middle, 2.25–3.20%; bottom, 1.75–2.81%) site during the period before meadow decline (Fig. 5). In contrast, no significant differences in the proportion of ABC transporters targeting sugars between sites were observed during the period of meadow decline. These transporters also showed a significant increase in the bottom layer of the nonvegetated site from the period before decline (1.75–2.81%) to the period of meadow decline (2.16–6.79%). The proportion of ABC transporters targeting amino acids only showed a significant increase in the bottom layer of the nonvegetated site from the period before decline (2.05–3.02%) to the period of meadow decline (2.76–4.62%).

**Fig. 6:**
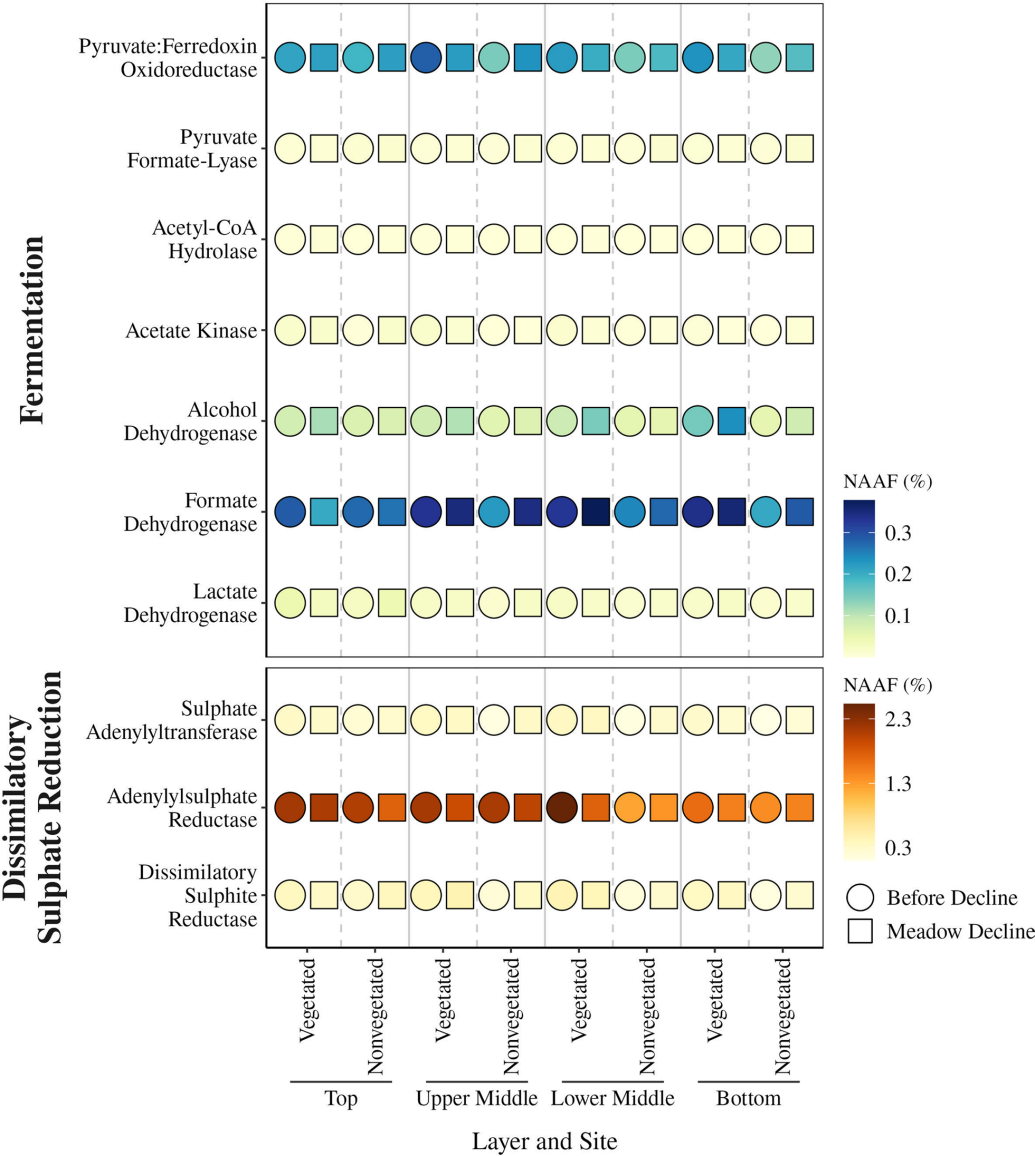
Median proportion of enzymes involved in mediating various fermentation products and dissimilatory sulphate reduction in sediment layers at the vegetated and nonvegetated site before and during the decline of the *C. nodosa* meadow in the Bay of Saline. The proportion was calculated using the NAAF

To evaluate the role of fermentation processes at these two sites, we selected enzymes from the KEGG database that are thought to be involved in mediating various fermentation products such as carbon dioxide, formate, acetate, acetone, ethanol, lactate, acetoin, propionate, and butyrate (Supplementary Table S4). Of these selected enzymes, our dataset contained pyruvate:ferredoxin oxidoreductase, pyruvate formate-lyase, acetyl-CoA hydrolase, acetate kinase, and alcohol, formate, and lactate dehydrogenase (Fig. 6). Formate dehydrogenase (45.2%), pyruvate:ferredoxin oxidoreductase (31.4%), and alcohol dehydrogenase (17.0%) were the most prominent of all fermentation-mediating enzymes detected. Significantly (*p* < 0.05) higher proportions of formate dehydrogenase were detected before meadow decline in the lower middle layer of the vegetated (0.28–0.45%) compared to the nonvegetated (0.20–0.30%) site. In contrast, no significant differences were observed during the decline. A similar trend was observed for pyruvate:ferredoxin oxidoreductase in the bottom layer, which had a higher proportion of this enzyme before the decline at the vegetated (0.14–0.30%) compared to the nonvegetated (0.09–0.22%) site. However, no significant differences between sites were observed during the decline (Fig. 6). Alcohol dehydrogenase showed significant differences between sites in both the lower middle and bottom layer. In the lower middle layer, a higher proportion of this enzyme was observed at the vegetated than at the nonvegetated site before (vegetated, 0.08–0.16%; nonvegetated, 0.04–0.07%) and during meadow decline (vegetated, 0.07–0.43%; nonvegetated, 0.04–0.11%). The same pattern of higher proportions of this enzyme at the vegetated site before (vegetated, 0.08–0.26%; nonvegetated, 0.05–0.07%) and during meadow decline (vegetated, 0.13–0.88%; nonvegetated, 0.06–0.14%) was also detected in the bottom layer.

To obtain an overview of the different microbial metabolic processes occurring in the sediment of the Bay of Saline, we selected KEGG modules describing methane-, nitrogen-, and sulphur-related processes (Supplementary Table S5). Among all tested modules we found proteins involved in the following processes: methanogenesis, nitrogen fixation, dissimilatory nitrate reduction, denitrification, assimilatory and dissimilatory sulphate reduction, and thiosulphate oxidation by the SOX complex. Since one of the most prominent proteins in the functional COG category C (energy production and conversion) was the adenylylsulphate reductase (Fig. 4), which is involved in dissimilatory sulphate reduction, the dynamics of the enzymes involved in this process were investigated in more detail (Supplementary Table S6). Of the enzymes involved in dissimilatory sulphate reduction, our dataset contained sulphate adenylyltransferase, adenylylsulphate reductase, and dissimilatory sulphite reductase (Fig. 6). The proportion of adenylylsulphate reductase was much higher (75.0%) than that of sulphate adenylyltransferase (11.1%) and dissimilatory sulphite reductase (13.9%). Significantly (*p* < 0.05) higher proportions of sulphate adenylyltransferase were observed before meadow decline at the vegetated than at the nonvegetated site in the upper middle (vegetated, 0.18–0.55%; nonvegetated, 0.10–0.28%), lower middle (vegetated, 0.27–0.61%; nonvegetated, 0.06–0.19%), and bottom (vegetated, 0.17–0.32%; nonvegetated, 0.06–0.20%) layer. In addition, significantly higher proportions were also found in the bottom layer of the nonvegetated site during decline (0.14–0.35%) compared to the period before meadow decline (0.06–0.20%). Proportions of the adenylylsulphate reductase showed significant changes in the lower middle layer, where higher values were observed before meadow decline at the vegetated (1.99–3.05%) compared to the nonvegetated (1.22–2.53%) site. Also, in the same layer of the vegetated site significantly higher proportions were detected before decline (1.99–3.05%) compared to the period of meadow decline (1.33–2.40%). In the lower middle and bottom layer, dissimilatory sulphite reductase showed higher proportions before meadow decline at the vegetated (lower middle, 0.37–0.78%; bottom, 0.25–0.42%) than at the novegetated (lower middle, 0.16–0.40%; bottom, 0.11–0.21%) site. In addition, significantly higher proportions were detected in the bottom layer of the nonvegetated site during decline (0.17–0.39%) compared to the period before meadow decline (0.11–0.21%). In contrast, no significant differences between sites were observed during the meadow decline for either of these enzymes (Fig. 6).

## Discussion

Seagrass meadow habitats are highly productive ecosystems [82] that support high biodiversity [83]. In the present study, before the decline of the *C. nodosa* meadow, higher values of the number of observed proteins and of the exponential of the Shannon diversity index were found in the vegetated compared to the nonvegetated sediment. These differences were more pronounced in the deeper parts of the sediment (i.e. in the lower middle and bottom layer). During meadow decline, the differences began to disappear and the values of the number of observed proteins and of the Shannon diversity index were similar to those observed in the sediment previously inhabited by the meadow. In addition, the structure of the metabolic profile of the communities inhabiting the nonvegetated sediment showed a separation between the period before and during meadow decline, especially in the deeper parts of the sediment and when only proteins for energy production and conversion were considered. This pattern was not observed for the communities at the vegetated site. The difference between the microbial communities inhabiting the vegetated and nonvegetated sediment in the period before meadow decline is not surprising, as several studies have found that the presence of seagrass leads to the formation of sediment communities that differ in composition [16–22] and function [15] from communities inhabiting nonvegetated sediments. In addition, the higher values of the number of observed proteins and of the Shannon diversity index at the vegetated site before the decline are consistent with other studies reporting higher metabolic diversity and microbial community activity in seagrass sediments compared to nonvegetated areas and with higher organic matter content in seagrass-inhabited sediments [11, 15]. In addition, the greater differentiation observed in the deeper layers in terms of protein richness and diversity is consistent with a previous study on the same sediment communities, which found a greater community separation between sites in the deeper parts of the sediment [21]. In contrast, the less pronounced differentiation observed for the same parameters and for the structure of the metabolic profile in the top and upper middle layer could be explained by the input of organic matter derived from the vegetated site, making the communities in the upper part of the sediment more similar to each other. Indeed, organic matter imported from the seagrass meadow has been shown to be an important source for prokaryotes in nonvegetated sediments [84].

The lack of differences between sites during the meadow decline in the number of observed proteins and in the exponential of the Shannon diversity index indicates the presence of a more uniform microbial metabolic profile during this period, similar to the metabolic profile observed at the vegetated site prior to decline. This observation is supported by the greater similarity in the structure of the metabolic profile of the communities at the nonvegetated site during decline with the metabolic profile of the communities in the upper sediment prior to decline. Because seagrass meadows fix the sediment by reducing resuspension rates and sediment mixing [85], we hypothesise that resuspension, mixing, and transport between sites are enhanced when the meadow is no longer present, allowing greater input of fresh organic matter to the nonvegetated sediment. Indeed, Najdek et al. (2020) [39] reported higher levels of total lipids and organic matter at the nonvegetated site during the *C. nodosa* decline from May to August 2018. The uniformity of the microbial profile observed at the vegetated site during the study could be the result of maintaining the source of organic matter during the decline of the seagrass through the decay of leaves, roots, and rhizomes [11–14].

The analysis of the functional COG categories showed that category C, which includes proteins for energy production and conversion, was the most abundant. This is consistent with the metagenomic study of Habibi et al. (2023) [86], who reported that energy production and conversion was also one of the most abundant functional COG categories in coastal sediments. Among these proteins, F-type H^+^-transporting ATPase subunit c, K^+^-stimulated pyrophosphate-energised sodium pump, and adenylylsulphate reductase subunits A and B exhibited the highest proportion. The pronounced presence of the F-type H^+^-transporting ATPase subunit c in the deeper parts of the nonvegetated sediment prior to decline could be explained by the involvement of this enzyme in the generation of membrane potential. The F-type ATPase can work in both directions, utilising the proton gradient to generate ATP or hydrolysing the ATP to generate the membrane potential [87]. The high proportion of the K^+^-stimulated pyrophosphate-energised sodium pump in our dataset indicates the coupling of the energy released by pyrophosphate hydrolysis with the active transport of cations across membranes [88, 89]. The proportion of this protein was increased in the deeper parts of the nonvegetated sediment during meadow decline, reflecting the need of microbial communities for more active cross-membrane transport probably due to the increased input of fresh organic matter from the vegetated site. The high proportion of adenylylsulphate reductase subunits A and B among the proteins for energy production and conversion is not surprising, as this enzyme is part of dissimilatory sulphate reduction to sulphide, a predominant terminal pathway of organic matter mineralisation in anoxic seabeds, where it reduces adenosine-5′-phosphosulphate to sulphite [90]. Furthermore, its significantly higher proportion in the nonvegetated sediment during decline could also be explained by the enhancement of this terminal pathway as a result of increased input of fresh organic matter from the vegetated site.

High molecular weight organic matter in marine sediments must be converted into low molecular weight molecules by various hydrolytic enzymes so that it can be taken up by cells [10]. Important components of organic matter in coastal marine sediments are carbohydrates, proteins, and lipids [91]. In our dataset, CAZymes and peptidases were more abundant than lipases, which may indicate the importance of carbohydrates and proteins as sources of organic matter for the microbial community. Among the CAZymes, the glycoside hydrolase families GH5 and GH9 were the most abundant. These families contain members capable of hydrolysing plant organic matter such as cellulose [92–94]. The presence of enzymes acting on cellulose is not surprising as cellulose is a major component of seagrass cell walls and contributes between 20 and 77% to the dry plant material [95–97]. Metalloendopeptidases and serine endopeptidases made up the vast majority of peptidases in our dataset. A high proportion of these enzymes among the extracellular proteases has already been reported for coastal sediments [98–100]. CAZymes and peptidases showed no dynamics from pre-decline to meadow decline, except that CAZymes decreased in the top layer of the nonvegetated sediment and peptidases increased in the upper middle layer of the vegetated sediment. Studies have shown that the molar carbon:nitrogen content in seagrass litter decreases during the decomposition process, which could be explained by increased microbial colonisation of detrital matter and microbial utilisation of exogenous nitrogen which could be related to the observed decrease in CAZymes and increase in peptidases [12, 14].

Once complex organic compounds have been broken down, the hydrolytic products can be transported into the cells. Prokaryotes utilise various transport proteins, including ABC transporters, to import substrates. Our metaproteomic dataset contained a large amount of ABC transporters for sugars and amino acids. Previous studies of sediment metagenomes also reported a high proportion of ABC transporters [86, 101]. The dynamics of ABC transporters reflected the overall dynamics of the metabolic profile. ABC transporters for sugars showed a higher proportion in the deeper parts of the vegetated compared to the nonvegetated sediment before meadow decline and showed no differences between sites during the decline, reflecting the higher organic matter content and corresponding demand for ABC transporters in seagrass-inhabited sediments [11, 15]. In addition, ABC transporters for sugars and amino acids showed an increase in their proportion in the bottom layer of the nonvegetated sediment from the period before decline to the period of meadow decline, which could be attributed to changes in organic matter content during the decline of the meadow.

The hydrolytic products of the degradation of complex organic matter, such as simple sugars and amino acids, can be imported and consumed by fermenting microbes. We have identified several enzymes involved in mediating various fermentation products, of which formate dehydrogenase, pyruvate:ferredoxin oxidoreductase, and alcohol dehydrogenase are the most abundant. Microcosm studies of the anaerobic degradation of organic matter in marine sediments revealed that acetate, formate, and ethanol are the most common fermentation products [102, 103]. In addition, direct measurements of sediment pore water have revealed the presence of methanol and ethanol [104]. It is therefore not surprising that the most common fermentation-mediating enzymes we found are putatively involved in the metabolism of acetate, formate, and ethanol. In addition, pyruvate:ferredoxin oxidoreductase and alcohol dehydrogenase have also been reported to be important fermentation-mediating enzymes in Baltic Sea sediments [105]. Similar to the dynamics of the ABC transporters, the fermentation-mediating enzymes also reflected the overall dynamics of the metabolic profile. Formate dehydrogenase, pyruvate:ferredoxin oxidoreductase, and alcohol dehydrogenase showed increased proportions in the deeper parts of the vegetated compared to the nonvegetated sediment before meadow decline, reflecting the higher organic matter content and possibly a higher fermentation rate in seagrass-inhabited sediments [11, 15].

The final step in the anaerobic degradation of organic matter involves the utilisation of simpler compounds such as SCFAs, including acetate and formate, and alcohols by the sulphate-reducing bacteria or methanogens [7, 9]. One of the most prominent proteins in functional COG category C was adenylylsulphate reductase, which is involved in dissimilatory sulphate reduction (Fig. 6). Dissimilatory sulphate reduction is known to be a predominant terminal pathway of organic matter mineralisation in anoxic seabeds [90]. Sulphate adenylyltransferase (Sat), adenylylsulphate reductase (Apr), and dissimilatory sulphite reductase (Dsr), enzymes involved in the dissimilatory sulphate reduction pathway shared by known sulphate-reducing microorganisms [90], were also detected in our metaproteomic dataset. The dynamics of these enzymes showed some common patterns that were comparable to the overall dynamics of the metabolic profile. Overall, higher proportions of these enzymes were found in the deeper parts of the vegetated compared to the nonvegetated site before the meadow decline, while such differences were not present during decline. This pattern as well as the overall dynamics of the metabolic profile could be explained by the higher organic matter content in the seagrass-inhabited sediments before decline [11, 15] and by the increased input of fresh organic matter into the nonvegetated area during meadow decline, probably as a result of increased resuspension rates and sediment mixing [85].

Metaproteomics has great potential to provide insights into the microbial response to environmental change in marine systems [24]. In the present study, metaproteomic analysis of MS/MS spectra using sequenced metagenomes from a subset of the same samples led to the identification of 57,305 proteins, more than in other metaproteomic studies of marine sediments [26, 28–30, 106]. Using this approach, it was possible to assess the dynamics of the metabolic profile of microbial communities in the sediment of a declining *C. nodosa* meadow and the surrounding nonvegetated sediment. Consequently, it was possible to assess the impact of seagrass decline on the metabolic profile of these communities.

## Conclusions

Seagrass sediments are considered natural hotspots for carbon sequestration, as some estimates suggest that up to 20% of global carbon sequestration in marine sediments occurs in these carbon-rich sediments, although they occupy only 0.1% of the seafloor [107–109]. Due to the role of seagrasses in carbon sequestration and their decline observed worldwide [31, 33–39], it is important to gain knowledge about the influence of this phenomenon on the processes carried out by the microbial community in the sediment.

The results of the present study show that the differences in the metabolic profile between the microbial communities inhabiting the vegetated and nonvegetated sediment, observed in the period before the meadow decline, disappeared during the decline. In addition, the metabolic profile of the nonvegetated communities approached that of the vegetated communities during the decline of the meadow. This phenomenon was more pronounced in the deeper parts of the nonvegetated sediment, indicating a stronger influence of the seagrass decline on the metabolic profile of the communities in this sediment layer. The differentiation between the vegetated and nonvegetated sediment when the meadow was present and the stronger shift in the profile observed in the deeper parts of the nonvegetated sediment during the decline could be explained by the intensity of the input of organic matter derived from the seagrass [11, 15]. The presence of the seagrass meadow reduces the intensity of resuspension, mixing, and transport between sites [85], thereby reducing the input of organic matter to the areas with bare sediment. However, with the decline of the seagrass, these processes are likely to be more intense, allowing organic matter to spread more easily throughout the area.

While these results provide a valuable foundation for future research, the relatively short sampling duration may not be sufficient to observe the full extent of microbial community response to seagrass decline, emphasising the need for longer-term studies. Furthermore, additional studies on microbial communities inhabiting sediments influenced by other seagrass species are needed to confirm the generalisability of the results reported in this study.

## Additional file

Supplementary file 1 (pdf 55 KB)

## Data Availability

The raw metagenomic sequences obtained in this study have been deposited in the European Nucleotide Archive (ENA) at EMBL-EBI under the accession number PRJEB75905 (https://www.ebi.ac.uk/ena/browser/view/PRJEB75905). The mass spectrometry proteomics data have been deposited in the ProteomeXchange Consortium via the PRIDE [110] partner repository with the dataset identifier PXD054602. Following the recommendations given from the Riffomonas project to enhance data reproducibility (http://www.riffomonas.org), the detailed analysis procedure, including the R Markdown file for this article, is available as a GitHub repository (https://github.com/MicrobesRovinj/Markovski_SalineSedimentMetap_EnvironMicrobiome_2025).
