# Supplementary material for: Shift in the metabolic profile of sediment microbial communities during seagrass decline
Source: Environ Microbiome. 2025 Jul 22;20:93. doi: 10.1186/s40793-025-00750-1 (PMC12281803; doi:10.1186/s40793-025-00750-1)
Supplement: Supplementary file 1 — (pdf 55 KB) [file 40793_2025_750_MOESM1_ESM.pdf]

Supplementary material

Shift in the metabolic profile of sediment microbial communities during seagrass decline

Marsej Markovski[1], Mirjana Najdek[1], Zihao Zhao[2], Gerhard J. Herndl[2,3], and Marino Korlević[1*]

1. Centre for Marine Research, Ruđer Bošković Institute, Croatia
2. Department of Functional and Evolutionary Ecology, University of Vienna, Austria
3. Department of Marine Microbiology and Biogeochemistry, Royal Netherlands Institute for Sea Research (NIOZ), Utrecht University, The Netherlands

[*]To whom correspondence should be addressed:

Marino Korlević

G. Paliaga 5, 52210 Rovinj, Croatia

Tel.: +385 52 804 768

e-mail: marino.korlevic@irb.hr

Supplementary tables

Supplementary Table S1 Sample ID, sampling date and site, and sediment depth and layer for each protein sample. Samples for which no MS/MS spectra were obtained are indicated with an asterisk.

Sample ID	Date	Site	Sediment Depth (cm)	Sediment Layer
MM_1	12 July 2017	Nonvegetated	0 – 1	Top
MM_2			2 – 3	Upper Middle
MM_3			4 – 5	Lower Middle
MM_4			7 – 8	Bottom
MM_5	12 July 2017	Vegetated	0 – 1	Top
MM_6			2 – 3	Upper Middle
MM_7			4 – 5	Lower Middle
MM_8			7 – 8	Bottom
MM_9	9 August 2017	Nonvegetated	0 – 1	Top
MM_10			2 – 3	Upper Middle
MM_11			4 – 5	Lower Middle
MM_12			7 – 8	Bottom
MM_13	9 August 2017	Vegetated	0 – 1	Top
MM_14			2 – 3	Upper Middle
MM_15			4 – 5	Lower Middle
MM_16			7 – 8	Bottom
MM_17	14 September 2017	Nonvegetated	0 – 1	Top
MM_18			2 – 3	Upper Middle
MM_19			4 – 5	Lower Middle
MM_20			7 – 8	Bottom
MM_21	14 September 2017	Vegetated	0 – 1	Top
MM_22			2 – 3	Upper Middle
MM_23			4 – 5	Lower Middle
MM_24			7 – 8	Bottom
MM_25	11 October 2017	Nonvegetated	0 – 1	Top
MM_26			2 – 3	Upper Middle
MM_27			3 – 4	Lower Middle
MM_28			7 – 8	Bottom
MM_29	11 October 2017	Vegetated	0 – 1	Top
MM_30			2 – 3	Upper Middle
MM_31			3 – 4	Lower Middle
MM_32			7 – 8	Bottom
MM_33	22 November 2017	Nonvegetated	0 – 1	Top
MM_34			2 – 3	Upper Middle
MM_35			4 – 5	Lower Middle
MM_36			7 – 8	Bottom
MM_37	22 November 2017	Vegetated	0 – 1	Top
MM_38			2 – 3	Upper Middle
MM_39			4 – 5	Lower Middle
MM_40			7 – 8	Bottom

Supplementary Table S1 Sample ID, sampling date and site, and sediment depth and layer for each protein sample. Samples for which no MS/MS spectra were obtained are indicated with an asterisk. *(continued)*

Sample ID	Date	Site	Sediment Depth (cm)	Sediment Layer
MM_41	13 December 2017	Nonvegetated	0 – 1	Top
MM_42			2 – 3	Upper Middle
MM_43			4 – 5	Lower Middle
MM_44			7 – 8	Bottom
MM_45	13 December 2017	Vegetated	0 – 1	Top
MM_46			2 – 3	Upper Middle
MM_47			4 – 5	Lower Middle
MM_48			7 – 8	Bottom
MM_49	12 February 2018	Nonvegetated	0 – 1	Top
MM_50			2 – 3	Upper Middle
MM_51			4 – 5	Lower Middle
MM_52			7 – 8	Bottom
MM_53	12 February 2018	Vegetated	0 – 1	Top
MM_54			2 – 3	Upper Middle
MM_55			4 – 5	Lower Middle
MM_56			7 – 8	Bottom
MM_57*	26 March 2018	Nonvegetated	0 – 1	Top
MM_58			2 – 3	Upper Middle
MM_59			5 – 6	Lower Middle
MM_60			7 – 8	Bottom
MM_61	26 March 2018	Vegetated	0 – 1	Top
MM_62			2 – 3	Upper Middle
MM_63*			5 – 6	Lower Middle
MM_64			7 – 8	Bottom
MM_65	23 April 2018	Nonvegetated	0 – 1	Top
MM_66			2 – 3	Upper Middle
MM_67			4 – 5	Lower Middle
MM_68			7 – 8	Bottom
MM_69	23 April 2018	Vegetated	0 – 1	Top
MM_70			2 – 3	Upper Middle
MM_71			4 – 5	Lower Middle
MM_72			7 – 8	Bottom
MM_73	21 May 2018	Nonvegetated	0 – 1	Top
MM_74			2 – 3	Upper Middle
MM_75			3 – 4	Lower Middle
MM_76			7 – 8	Bottom
MM_77	21 May 2018	Vegetated	0 – 1	Top
MM_78			2 – 3	Upper Middle
MM_79			3 – 4	Lower Middle
MM_80			7 – 8	Bottom

Supplementary Table S1 Sample ID, sampling date and site, and sediment depth and layer for each protein sample. Samples for which no MS/MS spectra were obtained are indicated with an asterisk. *(continued)*

Sample ID	Date	Site	Sediment Depth (cm)	Sediment Layer
MM_81	18 June 2018	Nonvegetated	0 – 1	Top
MM_82			2 – 3	Upper Middle
MM_83			4 – 5	Lower Middle
MM_84			7 – 8	Bottom
MM_85	18 June 2018	Vegetated	0 – 1	Top
MM_86			2 – 3	Upper Middle
MM_87			4 – 5	Lower Middle
MM_88			7 – 8	Bottom
MM_89	9 July 2018	Nonvegetated	0 – 1	Top
MM_90			2 – 3	Upper Middle
MM_91			4 – 5	Lower Middle
MM_92			7 – 8	Bottom
MM_93	9 July 2018	Vegetated	0 – 1	Top
MM_94			2 – 3	Upper Middle
MM_95			4 – 5	Lower Middle
MM_96			7 – 8	Bottom
MM_97	8 August 2018	Nonvegetated	0 – 1	Top
MM_98			2 – 3	Upper Middle
MM_99			4 – 5	Lower Middle
MM_100			7 – 8	Bottom
MM_101	8 August 2018	Vegetated	0 – 1	Top
MM_102			2 – 3	Upper Middle
MM_103			4 – 5	Lower Middle
MM_104			7 – 8	Bottom
MM_105	3 September 2018	Nonvegetated	0 – 1	Top
MM_106			2 – 3	Upper Middle
MM_107			4 – 5	Lower Middle
MM_108			7 – 8	Bottom
MM_109	3 September 2018	Vegetated	0 – 1	Top
MM_110			2 – 3	Upper Middle
MM_111			4 – 5	Lower Middle
MM_112			7 – 8	Bottom
MM_113	4 October 2018	Nonvegetated	0 – 1	Top
MM_114			2 – 3	Upper Middle
MM_115			4 – 5	Lower Middle
MM_116			7 – 8	Bottom
MM_117	4 October 2018	Vegetated	0 – 1	Top
MM_118			2 – 3	Upper Middle
MM_119			4 – 5	Lower Middle
MM_120			7 – 8	Bottom

Supplementary Table S2 Sample ID, sampling site, sediment layer and depth, sampling date, number of raw sequence pairs, number of assembled contigs by MEGAHIT, N50 and L50 assembly statistics, number of predicted CDSs by Prodigal, and number of eggNOG-mapper annotated CDSs.

Sample ID	Site	Layer (Depth)	Date	No. of Raw Sequence Pairs	No. of Contigs	N50*	L50 (bp)*	No. of Predicted CDSs	No. of Annotated CDSs
356	Vegetated	Top (0 – 1 cm)	8 August 2018	205,085,833	32,026,408	8,760,379	601	40,693,178	29,364,186
358		Lower Middle (4 – 5 cm)		209,632,803	33,248,196	9,111,820	590	42,249,295	29,892,039
360	Nonvegetated	Top (0 – 1 cm)	8 August 2018	213,766,540	21,634,340	6,073,512	595	27,526,969	19,599,377
362		Lower Middle (4 – 5 cm)		216,556,629	27,534,653	8,174,204	592	34,788,216	24,307,842

* The notation was preserved from the original output of BBTools statswrapper.sh.

Supplementary Table S3 The proportion of each COG functional category (NAAF) and the number of proteins assigned to each category. The proportion and the number of proteins assigned to category C (energy production and conversion) are highlighted.

COG Category	NAAF (%)	Number of Proteins
**C – Energy production and conversion**	**15.18**	**8,224**
S – Function unknown	12.62	6,299
G – Carbohydrate transport and metabolism	11.45	6,823
E – Amino acid transport and metabolism	9.25	6,893
M – Cell wall/membrane/envelope biogenesis	8.89	2,999
P – Inorganic ion transport and metabolism	8.43	4,441
Multiple functional categories	7.65	2,603
O – Posttranslational modification, protein turnover, chaperones	7.02	2,901
J – Translation, ribosomal structure and biogenesis	5.28	1,507
H – Coenzyme transport and metabolism	2.29	1,494
Q – Secondary metabolites biosynthesis, transport and catabolism	2.26	1,356
F – Nucleotide transport and metabolism	1.78	1,475
I – Lipid transport and metabolism	1.72	1,421
N – Cell motility	1.38	599
K – Transcription	1.35	1,169
U – Intracellular trafficking, secretion, and vesicular transport	1.27	731
L – Replication, recombination and repair	0.98	397
T – Signal transduction mechanisms	0.79	574
V – Defense mechanisms	0.23	217
D – Cell cycle control, cell division, chromosome partitioning	0.18	147
Total	100.00	52,270

Supplementary Table S4 Overview of selected enzymes, their enzymatic products, and corresponding KO entries used to evaluate mediation processes of various fermentation products.

Name	Product	KO Entry
Pyruvate:ferredoxin oxidoreductase	Acetyl-CoA, carbon dioxide	K00169<br>K00170<br>K00171<br>K00172<br>K03737
Pyruvate formate-lyase	Acetyl-CoA, formate	K00656
Acetyl-CoA hydrolase	Acetate	K01067
Acetate kinase	Acetate	K00925
Acetoacetate decarboxylase	Acetone, carbon dioxide	K01574
Alcohol dehydrogenase	Ethanol	K00001<br>K04022<br>K13951<br>K13952<br>K13954<br>K13980<br>K18857<br>K00002<br>K13979<br>K00114
Formate dehydrogenase	Carbon dioxide	K00122<br>K00123<br>K00124<br>K00126<br>K00127<br>K22515<br>K05299<br>K15022<br>K00125<br>K22516
Lactate dehydrogenase	Lactate	K00016
Acetolactate decarboxylase	Acetoin, carbon dioxide	K01575
Methylmalonyl-CoA decarboxylase	Propionyl-CoA, carbon dioxide	K11264<br>K01604
Lactoyl-CoA dehydratase	Acryloyl-CoA	K20626<br>K20627
Propionaldehyde dehydrogenase	Propionyl-CoA	K13922
Butyrate kinase	Butyrate	K00929
Butyryl-CoA:acetate CoA transferase	Butyrate, acetyl-CoA	K01034<br>K01035

Supplementary Table S5 Overview of KEGG modules used for assessing various types of microbial metabolism.

Type of Metabolism	KEGG Module
Methanogenesis	M00567<br>M00357<br>M00356<br>M00563
Methane oxidation	M00174
Nitrogen fixation	M00175
Assimilatory nitrate reduction	M00531
Dissimilatory nitrate reduction	M00530
Denitrification	M00529
Nitrification	M00528
Complete nitrification, comammox	M00804
Anammox	M00973
Assimilatory sulphate reduction	M00176
Dissimilatory sulphate reduction	M00596
Thiosulfate oxidation by SOX complex	M00595

Supplementary Table S6 Enzymes involved in dissimilatory sulphate reduction and their KO entries.

Name	KO Entry
Sulphate adenylyltransferase	K00958
Adenylylsulphate reductase	K00394<br>K00395
Dissimilatory sulphite reductase	K11180<br>K11181<br>K27196
